# Spine surgeon perceptions of the challenges and benefits of telemedicine: an international study

**DOI:** 10.1007/s00586-020-06707-x

**Published:** 2021-01-16

**Authors:** Grant J. Riew, Francis Lovecchio, Dino Samartzis, David N. Bernstein, Ellen Y. Underwood, Philip K. Louie, Niccole Germscheid, Howard S. An, Jason Pui Yin Cheung, Norman Chutkan, Gary Michael Mallow, Marko H. Neva, Frank M. Phillips, Daniel M. Sciubba, Mohammad El-Sharkawi, Marcelo Valacco, Michael H. McCarthy, Sravisht Iyer, Melvin C. Makhni

**Affiliations:** 1 Harvard Medical School, Boston, MA USA; 2 Department of Orthopaedic Surgery, Harvard Medical School, Brigham and Women’s Hospital, Boston, MA USA; 3 Department of Orthopaedic Surgery, Hospital for Special Surgery, New York, NY USA; 4 Department of Orthopaedic Surgery, Rush University Medical Center, Chicago, IL USA; 5 The International Spine Research and Innovation Initiative, Rush University Medical Center, Chicago, IL USA; 6 Department of Anesthesiology, Critical Care and Pain Medicine, Boston Children’s Hospital, Harvard University, Boston, MA USA; 7 Virginia Mason Medical Center, Neuroscience Institute, Seattle, WA USA; 8 Research Department, AO Spine International, Davos, Switzerland; 9 Department of Orthopaedics and Traumatology, The University of Hong Kong, Pok Fu Lam, Hong Kong SAR China; 10 Department of Orthopaedic Surgery, University of Arizona College of Medicine, Phoenix, AZ USA; 11 Department of Orthopaedic and Trauma Surgery, Tampere University Hospital, Tampere, Finland; 12 Department of Neurosurgery, Johns Hopkins University, Baltimore, MD USA; 13 Department of Orthopaedic and Trauma Surgery, Assiut University Medical School, Assiut, Egypt; 14 Department of Orthopaedics, Churruca Hospital de Buenos Aires, Buenos Aires, Argentina; 15 Indiana Spine Group, Carmel, IN USA

**Keywords:** Telemedicine, Global, Spine surgery, Benefits, Challenges

## Abstract

**Introduction:**

While telemedicine usage has increased due to the COVID-19 pandemic, there remains little consensus about how spine surgeons perceive virtual care. The purpose of this study was to explore international perspectives of spine providers on the challenges and benefits of telemedicine.

**Methods:**

Responses from 485 members of AO Spine were analyzed, covering provider perceptions of the challenges and benefits of telemedicine. All questions were optional, and blank responses were excluded from analysis.

**Results:**

The leading challenges reported by surgeons were decreased ability to perform physical examinations (38.6%), possible increased medicolegal exposure (19.3%), and lack of reimbursement parity compared to traditional visits (15.5%). Fewer than 9.0% of respondents experienced technological issues. On average, respondents agreed that telemedicine increases access to care for rural/long-distance patients, provides societal cost savings, and increases patient convenience. Responses were mixed about whether telemedicine leads to greater patient satisfaction. North Americans experienced the most challenges, but also thought telemedicine carried the most benefits, whereas Africans reported the fewest challenges and benefits. Age did not affect responses.

**Conclusion:**

Spine surgeons are supportive of the benefits of telemedicine, and only a small minority experienced technical issues. The decreased ability to perform the physical examination was the top challenge and remains a major obstacle to virtual care for spine surgeons around the world, although interestingly, 61.4% of providers did not acknowledge this to be a major challenge. Significant groundwork in optimizing remote physical examination maneuvers and achieving legal and reimbursement clarity is necessary for widespread implementation.

## Introduction

Telemedicine usage among spine surgeons has rapidly risen in response to the novel coronavirus (COVID-19) pandemic and social distancing directives [1–6]. In a global survey investigating the impact of COVID-19 on spine surgeons, Louie et al*.* [1] found that 35.6% of respondents were performing the majority of appointments via telemedicine by the end of the first quarter of 2020. While the current necessity of remote clinical care is evident, little is known about how spine surgeons perceive the routine use of telemedicine.

Advocates of telemedicine describe widespread benefits that include: increased access to care, high clinician/patient satisfaction rates, and overall cost savings [7–9]. Opponents, on the other hand, are concerned about greater medicolegal exposure, decreased ability to perform physical examinations, and weaker doctor–patient relationships, thus highlighting the importance of in-person visits [10]. Since the beginning of the pandemic, research has shown high patient satisfaction rates with spine telemedicine; in a study of 772 patients, Satin et al. [11] found that 87.7% were satisfied with telemedicine and 45% preferred telemedicine over in-person visits if given the option. Additionally, pilot guidelines for suggested telemedicine physical examination maneuvers have been published, though there still remains little consensus for how to best conduct the remote examination [12, 13].

While several studies have been published regarding patient satisfaction and physical examination techniques, to our knowledge, throughout spine, orthopedic surgery, or other surgical subspecialties, no data-driven study has assessed the challenges and benefits of telemedicine from an international surgeon perspective. This global survey study sought to address this deficiency by analyzing the overall and regionally reported challenges and perceived benefits of telemedicine among spine surgeons, and investigating how additional factors, such as age, type of platform used, number of visits performed, and specialty, influence surgeons’ perspectives.

## Methods

### Survey design and distribution

Our survey was designed to assess provider experiences and perspectives about the challenges and benefits of telemedicine. Using a Delphi approach, a group of board-certified spine surgeons, research representatives, and epidemiologists developed a comprehensive 42-question survey including questions covering demographics, telemedicine usage, provider perceptions, telemedicine challenges and benefits, and telemedicine in research and teaching.

The survey, titled “Telemedicine and the Spine Surgeon—Perspectives and Practices Worldwide,” was distributed by email to the 3805 members of AO Spine who elected to be subscribed to all emails. Surgeons were given from May 15 to May 31, 2020, to respond. All questions were optional, and missing data were excluded from the analysis.

### Statistical analyses and survey interpretation

All statistical analyses were performed with SPSS version 25 (IBM Corp., Armonk, NY). Tables and graphical representation of survey responses were performed using Excel version 16.37 (Microsoft Inc, Albuquerque, NM) and the open-source Python “plotly” library (version 4.8.2). Descriptive statistics were used to describe overall and regional responses, and differences were compared across region. Differences in responses were also compared among age (< 45 years vs. ≥ 45 years), platform (video vs audio), visits (≤ 50 vs. > 50), and specialty (orthopedic surgery vs. neurosurgery). Categorical variables were compared using Chi-squared tests. Likert scale questions were analyzed as continuous variables with ANOVA and Mann–Whitney U tests as appropriate. The following Likert scale was used: − 2 strongly disagree;  − 1 disagree; 0 neutral; and 1 agree; 2 strongly agree. The threshold for statistical significance was set at *p* < 0.05.

## Results

### Respondent overview (Fig. 1)

**Fig. 1:**
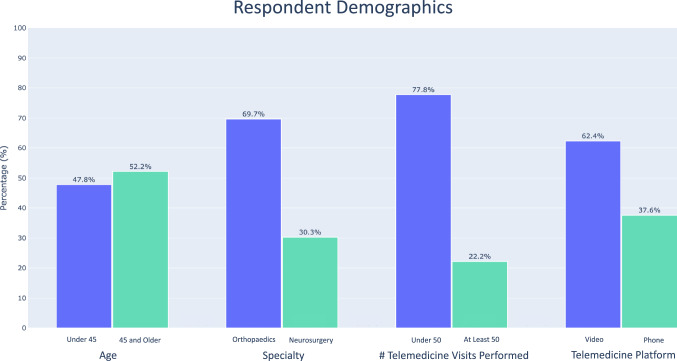
Demographic distribution of overall responses for age, platform, number of visits performed, and specialty

Overall, 485 surgeons responded to the survey from 75 different countries. Approximately half were under the age of 45 (229/479; 47.8%), while 250/479 (52.2%) were 45 or older. Most (166/266; 62.4%) performed video visits (secure EMR or nonsecure—i.e., FaceTime, Skype, etc.), versus audio-only phone encounters (100/266; 37.6%) as their main telemedicine platform. The majority specialized in orthopedic surgery (332/476; 69.7%) versus neurosurgery (144/476; 30.3%) (respondents could select more than one specialty, and “trauma” (50 responses), “pediatric surgery” (16 responses), and “other” (14 responses) were excluded from analysis).

### Challenges (Table 1)

**Table 1:** Challenges experienced by provider and patient when delivering care via telemedicine

	Overall		Africa		Asia Pacific		Europe		North America		South America		*p* value^a^
	*n* ^b^	% Total	*n* ^b^	% Region	*n* ^b^	% Region	*n* ^b^	% Region	*n* ^b^	% Region	*n* ^b^	% Region	
**Provider experienced challenges**													
Lack of access to Internet	40	8.4	10	10.5	11	11.7	8	6.9	3	6.7	8	6.3	0.535
Lack of access to computer/phone with camera	30	6.3	1	1.1	6	6.4	13	11.2	3	6.7	7	5.5	0.054
Lack of access to telephone	20	4.2	2	2.1	4	4.3	9	7.8	1	2.2	4	3.1	0.241
Lack of technological literacy	36	7.5	5	5.3	11	11.7	7	6.0	6	13.3	7	5.5	0.176
Technology implementation and maintenance costs	24	5.0	4	4.2	7	7.4	2	1.7	2	4.4	9	7.1	0.278
Decreased ability to perform physical examinations	184	38.6	25	26.3	39	41.5	48	41.4	28	62.2	44	34.6	**< 0.001**
Possible increased medicolegal exposure	92	19.3	16	16.8	12	12.8	25	21.6	12	26.7	27	21.3	0.267
Lack of reimbursement parity versus traditional visits	74	15.5	11	11.6	12	12.8	17	14.7	14	31.1	20	15.7	**0.039**
Unclear billing codes	53	11.1	5	5.3	11	11.7	8	6.9	15	33.3	14	11.0	**< 0.001**
Regulatory barriers	48	10.1	4	4.2	9	9.6	14	12.1	9	20.0	12	9.4	0.059
Other	18	3.8	1	1.1	5	5.3	6	5.2	2	4.4	4	3.1	0.498
**Patient experienced challenges**													
Lack of access to Internet	84	17.6	15	15.8	22	23.4	16	13.8	13	28.9	18	14.2	0.074
Lack of access to computer/phone with camera	85	17.8	13	13.7	14	14.9	14	12.1	19	42.2	25	19.7	**< 0.001**
Lack of access to telephone	33	6.9	4	4.2	8	8.5	10	8.6	4	8.9	7	5.5	0.621
Lack of technological literacy	115	24.1	16	16.8	25	26.6	26	22.4	21	46.7	27	21.3	**0.003**
Perceived lack of privacy	43	9.0	8	8.4	10	10.6	10	8.6	5	11.1	10	7.9	0.937
Concern over paying for care	57	11.9	10	10.5	17	18.1	6	5.2	7	15.6	17	13.4	0.052
Other	22	4.6	4	4.2	4	4.3	8	6.9	2	4.4	4.0	3.1	0.725

Bolded values indicate statistical significance at *p* < .05

^a^Calculation of *p* values was performed using Chi-squared tests.

^b^Number of respondents/votes

#### Provider experienced challenges

There were several challenges experienced by providers. The most common were: decreased ability to perform physical examinations (184/477; 38.6%), possible increased medicolegal exposure (92/477; 19.3%), and lack of reimbursement parity compared to traditional visits (74/477; 15.5%). Additional challenges providers faced included: unclear billing codes (53/477; 11.1%), regulatory barriers (48/477; 10.1%), lack of access to Internet (40/477; 8.4%), lack of technological literacy (36/477; 7.5%), lack of access to camera (30/477; 6.3%), technology implementation and maintenance costs (24/477; 5.0%), lack of access to telephone (20/477; 4.2%), and other (18/477; 3.8%).

Decreased ability to perform physical examinations (*p* < 0.001), lack of reimbursement parity (*p* = 0.039), and unclear billing codes (*p* < 0.001) exhibited regionally significant differences; North American providers described the greatest percentage of challenges in these specific topics with 62.2%, 31.1%, and 33.3% of surgeons noting these difficulties, respectively. Neither age nor number of visits performed significantly affected responses (Table 2). Surgeon specialty only affected the perception of reimbursement parity compared to traditional visits (*p* = 0.031), with 10.1% of neuro- and 18.1% of orthopedic surgeons having experienced this challenge.

**Table 2:** Influence of age, platform, visits performed, specialty on challenges, and benefits

	Age (< 45 vs. ≥ 45)	Platform (video vs. phone)	Visits performed (< 50 vs. ≥ 50)	Specialty (neuro vs. ortho)
**Provider experienced challenges**				
Lack of access to Internet	0.120	0.419	0.106	0.558
Lack of access to computer/phone with camera	0.207	0.096	0.602	0.319
Lack of access to telephone	0.668	** < 0.001**	0.109	0.739
Lack of technological literacy	0.942	0.191	0.732	0.109
Technology implementation and maintenance costs	0.537	0.771	0.493	0.184
Decreased ability to perform physical examinations	0.162	0.397	0.926	0.058
Possible increased medicolegal exposure	0.906	0.771	0.654	0.597
Lack of reimbursement parity vs. traditional visits	0.629	0.575	0.223	**0.031**
Unclear billing codes	0.599	0.706	0.139	0.109
Regulatory barriers	0.667	0.988	0.411	0.505
Patient experienced challenges (exclude from abstract, add in paper)				
Lack of access to Internet	0.106	**< 0.001**	**0.026**	0.189
Lack of access to computer/phone with camera	0.938	**0.002**	0.961	0.431
Lack of access to telephone	0.322	0.122	0.226	0.469
Lack of technological literacy	0.827	**0.002**	0.877	0.134
Perceived lack of privacy	0.858	0.534	0.304	0.217
Concern over paying for care received over telemedicine	0.621	0.621	**0.005**	0.171
**Perceived benefits of telemedicine**				
(− 2 strongly disagree, − 1 disagree, 0 neutral, 1 agree, 2 strongly agree)				
Telemedicine increases patient satisfaction	0.352	**0.047**	0.173	0.128
Telemedicine increases patient convenience	0.622	0.093	0.237	0.392
Telemedicine increases provider convenience	0.231	**0.019**	**0.012**	0.103
Telemedicine increases access to care for rural/international patients	0.840	0.076	0.610	0.576
Telemedicine decreases overhead for providers	0.342	0.160	0.770	0.901
Telemedicine provides cost savings (travel expenses, decreased hospital transfers, etc.)	0.352	0.144	0.132	0.473

Calculation of *p* values was performed using Pearson Chi-squared tests and Mann–Whitney *U* tests for Likert scale questions

Bolded values indicate statistical significance at *p* < .05

#### Perceived patient challenges

In order of frequency, the perceived challenges experienced by patients included lack of technological literacy (115/477; 24.1%), lack of patient access to camera (85/477; 17.8%), lack of patient access to Internet (84/477; 17.6%), concern for paying for care received over telemedicine (57/477; 11.9%), perceived lack of privacy (43/477; 9.0%), lack of access to telephone (33/477; 6.9%), and other (22/477; 4.6%). Perception of patient lack of access to camera (*p* < 0.001) and technological literacy (*p* = 0.003) varied significantly per region. Once again, surgeons in North America reported the highest rates of these perceived challenges, with 42.2% and 46.7% of physicians highlighting these concerns, respectively. Neither age nor specialty affected responses (Table 2). Those that performed > 50 telemedicine visits believed that patients had fewer issues with Internet access (22.6%, *p* = 0.026) and less concern for payment (9.4%, *p* = 0.005) compared to those that performed ≤ 50 visits (Internet issues: 39.2%; payment issues: 28.0%).

### Benefits (Table 3)

**Table 3:** Perceived benefits of telemedicine

(− 2 strongly disagree, − 1 disagree, 0 neutral, 1 agree, 2 strongly agree)	Overall		Africa		Asia Pacific		Europe		North America		South America		*p* Value^a^
	Mean	SD	Mean	SD	Mean	SD	Mean	SD	Mean	SD	Mean	SD	
Telemedicine increases patient satisfaction	0.06	0.90	− 0.23	0.80	− 0.12	0.93	0.05	0.86	0.48	0.87	0.13	0.91	**0.011**
Telemedicine increases patient convenience	0.69	0.88	0.45	0.68	0.73	0.93	0.54	0.89	1.30	0.77	0.60	0.87	**< 0.001**
Telemedicine increases provider convenience	0.34	0.94	− 0.07	0.77	0.38	0.98	0.31	0.82	0.66	1.18	0.40	0.92	**0.045**
Telemedicine increases access to care for rural/international patients (patients from long distance)	1.03	0.83	0.68	0.65	1.16	0.77	0.94	0.83	1.18	0.95	1.12	0.87	0.052
Telemedicine decreases overhead for providers	0.26	0.82	0.29	0.74	0.29	1.09	0.24	0.69	0.24	0.99	0.25	0.63	0.996
Telemedicine provides societal cost savings (travel expenses, decreased hospital transfers, etc.)	1.00	0.84	0.74	0.82	1.04	0.98	0.98	0.78	1.27	0.76	0.98	0.83	0.165

Bolded values indicate statistical significance at *p* < .05

^a^Calculation of *p* values was performed using ANOVA tests.

Overall, survey respondents agreed that telemedicine increases access to care for rural/long-distance patients (mean = 1.03; *SD* = 0.83), provides societal cost savings (mean = 1.00; *SD* = 0.84), and increases patient convenience (mean = 0.69; *SD* = 0.88). Providers slightly agreed that telemedicine increases provider convenience (mean = 0.34; *SD* = 0.94) and decreases overhead for providers (mean = 0.26; *SD* = 0.82). Notably, providers were neutral about whether telemedicine increases patient satisfaction (mean = 0.06; *SD* = 0.90). North Americans agreed the most with the benefits (patient satisfaction: mean = 0.48/*SD* = 0.87, *p* = 0.011; patient convenience: mean = 1.30/*SD* = 0.77, *p* < 0.001; provider convenience: mean = 0.66/SD = 1.18, *p* = 0.045) and Africans agreed the least (patient satisfaction: mean = − 0.23/*SD* = 0.80, *p* = 0.011; patient convenience: mean = 0.45/*SD* = 0.68, *p* < 0.001; provider convenience: mean = − 0.07/*SD* = 0.77, *p* = 0.045) (Table 3). Neither age nor specialty affected provider perceived benefits (Table 2). Physicians who performed video telemedicine visits believed patient satisfaction (mean = 0.16/*SD* = 0.92, *p* = 0.047) and provider convenience (mean = 0.46/*SD* = 0.94, *p* = 0.019) were higher compared to surgeons who performed audio-only phone call telemedicine visits (patient satisfaction: mean = − 0.09/*SD* = 0.86; provider convenience: mean = 0.16/*SD* = 0.95). Those that performed > 50 visits thought that telemedicine improved provider convenience (mean = 0.62/*SD* = 0.97, *p* = 0.012) more than those that performed ≤ 50 visits (mean = 0.26/*SD* = 0.92).

**Fig. 2:**
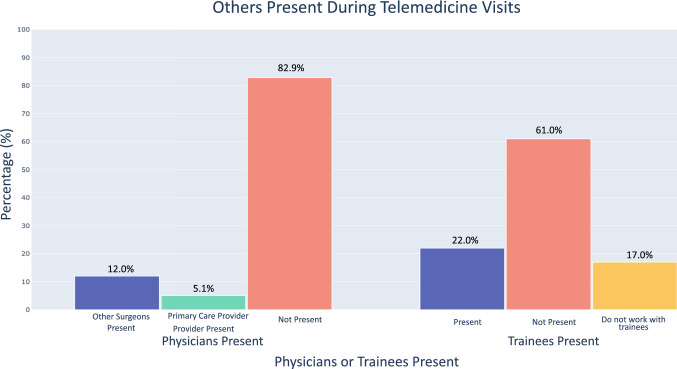
Others (providers or trainees) present during a telemedicine visit

The majority (180/217; 82.9%) of surgeons did not perform telemedicine with another surgeon present. Likewise, 170/218 (78.0%) of respondents either did not have trainees present during visits or did not work with trainees (Fig. 2).

## Discussion

To our knowledge, this is the first international study to assess physician-perceived challenges and benefits of telemedicine in spine surgery. Based on our survey, we found that telemedicine provides significant advantages in the current socially distanced environment. Recent evidence suggests that spine telemedicine is feasible [5, 11, 14], but there are doubts about whether telemedicine will continue to be a viable option once shelter-in-place demands subside due to provider preferences and patient demand [15]. We found that 95.4% of our respondents required one in-person visit prior to surgery and overwhelmingly favored in-person visits, echoing the concerns over whether clinicians will continue to offer telemedicine in the future. Our results provide insight into the pros and cons of telemedicine, which we hope will aid payers, hospital systems, administrators, researchers, and surgeons in determining whether and how to best integrate effective telehealth care after the pandemic.

### Challenges

Prior to COVID-19, it was thought that factors hindering mass adoption of telemedicine in spine surgery included unfamiliar learning curves, large technology costs, reimbursement difficulty, increased liability, and difficulty performing virtual physical examinations [10]. Our results showed that the most substantial challenge was the decreased ability to perform the physical examination, with nearly 40% of respondents highlighting this issue. Recent manuscripts have sought to address this challenge by publishing guidelines how to conduct effective virtual spine examinations [13, 16]. Additionally, efforts to enhance the telemedicine appointment—such as providing instructions for patients prior to the visit on camera/body positioning, clothing, and setting—have shown to increase telemedicine efficiency [17, 18]. While this inability to perform physical examinations was a major challenge faced by spine surgeons around the world, it is also interesting to note that 61.4% of survey respondents did not acknowledge the lack of physical examination to be a major challenge.

Regulatory frameworks had been confusing and rapidly changing at the outset of the pandemic. According to our survey, 10–20% of spine surgeons worried about increased medicolegal exposure or reimbursement parity. However, as time has elapsed, policies and laws around telemedicine have become more clear and standardized as more providers have shifted toward telemedicine [10, 19]. Additional guidelines and regulations are necessary as the field continues to evolve, as some of these in existence have only been temporary for the duration of the COVID-19 pandemic [20]. Notably, few providers had issues with technology—less than 9.0% of respondents noted problems with Internet, computers, or phones. However, patients appeared to struggle more frequently; 24.1% of surgeons reported that patient “lack of technological literacy” was an issue. While some studies have found that technology is a significant barrier to the telemedicine clinical workflow [21], others have found strikingly high success rates; Eichberg et al. [22] analysis of 52 neurosurgery studies found that telemedicine was successful in an astonishing 99.6% of cases. Moreover, with the increasing integration of telemedicine into EMR systems, proper training, and standardization of practices, technological difficulties may not be a major hindrance to effective spine telemedicine use.

### Benefits

Our survey suggests that spine surgeons believed that telemedicine carried certain benefits. Providers agreed that telemedicine can provide societal cost savings, consistent with cost analyses showing telemedicine can increase savings and decrease the need for unnecessary travel [23, 24]. Additionally, respondents agreed that telemedicine can enhance access to care for patients—for example those living in rural, developing, or resource-limited areas [25]. Interestingly, while surgeons slightly agreed that telemedicine increases patient and provider convenience, they were neutral to the statement that telemedicine increases patient satisfaction. Many studies have shown that telemedicine satisfaction is comparable to and sometimes exceeds that of in-person visits [22, 26, 27]. However, our data suggest that spine surgeons may not hold this view.

Prior telemedicine studies also examined the benefits of facilitating simultaneous communication between the patient and multiple providers during visits [28]. However, our study showed that few spine surgeons had other physicians (17.1%) or trainees (22.0%) present during telemedicine visits, signifying that the majority of providers are not utilizing this proposed advantage (Fig. 2). The feasibility of the multidisciplinary examination (providing simultaneous provider perspectives—general practitioner, surgeon, etc.) is clearly improved with virtual consults; however, improved coordination and collaborative logistics are necessary to integrate such visits into routine clinical care. Additionally, in the future, it may be important for more surgeons to consider including trainees to teach new surgeons how to properly provide telemedicine visits.

### Regional and respondent variation

North American providers encountered the most challenges, but also were the most optimistic about benefits. A multitude of North American studies has noted physical examinations, reimbursement parity, and unclear billing codes as barriers for telemedicine, but also has noted the potential benefits of increased patient/provider convenience and patient satisfaction [3, 8]; our study echoed this duality, underscoring the importance of addressing the shortcomings of telemedicine to sustain adoption. On the other hand, African respondents tended to downplay both challenges (billing codes, physical examination) and benefits (patient satisfaction/provider convenience). These providers may have a different perspective on telemedicine, prioritizing expansion of health access in rural settings and dealing with more underdeveloped settings [25, 29].

Although older individuals may be expected to experience more troubles with telemedicine [21], our study noted no differences in responses based on age-group. Providers who used audio-visual versus audio-only telemedicine had better opinions of telemedicine, understandably because the video component adds personalization and the ability to observe the patient. Surgeons that performed more visits (> 50 vs. ≤ 50) tended to also have higher opinions of telemedicine, noting fewer Internet and payment issues and appreciating the convenience of virtual visits more—reinforcing the idea that the more visits one performs, the more comfortable one becomes [5].

### Limitations

Our study represents an international perspective on the benefits and challenges of telemedicine. However, it has limitations inherent in a survey study. Approximately 12.5% of surgeons who were emailed responded, and because every question was optional, not every response had an answer. Additionally, each of the challenges and benefits were assessed from the provider perspective, as no patients were included in the survey. Finally, AO Spine may not be representative of all spine surgeons, either in its global or regional membership. Despite these limitations, 485 surgeons across the world responded to our survey, making this the largest international survey of spine surgeons that addressed the topic of telemedicine.

## Conclusion

To our knowledge, our study is the largest international initiative to assess spine surgeon perspectives on the challenges and benefits of telemedicine. Surgeons are aware of the benefits of increased access to care and cost savings. We also found that surgeons experienced fewer challenges than expected, with less than 9.0% reporting technical difficulties with telemedicine. Lack of a physical examination was found to be the most reported challenge in providing virtual care. However, 61.4% of respondents did not acknowledge this as an issue in delivering care via telemedicine, which could be a product of rapidly evolving methods to streamline remote patient assessment. Legal and reimbursement challenges were shown to be a significant concern. Fluctuating and unpredictable rules around telemedicine legalities and reimbursement rates stabilized significantly since the outset of the pandemic, and further clarity will likely arise as the world pushes to a post-COVID steady state. These barriers have limited telemedicine use and will hopefully be mitigated as permanent policies are enacted to regulate laws and reimbursements surrounding telemedicine. Given recent evidence attesting to the feasibility of telemedicine in spine surgery and promising patient interest, telemedicine may remain as a viable care option in the future. Ultimately, weighing the challenges and benefits of telemedicine may help determine whether spine surgeons continue to use telemedicine in the post-pandemic world.
